# Distinct Dietary Patterns Are Differentially Associated with Dyslipidemia Among Middle-Aged and Older Adults in Southern China

**DOI:** 10.3390/nu18193154

**Published:** 2026-09-24

**Authors:** Shilong Guo, Hailing Su, Miaolin Han, Yixi Zhang, Qiuyun Cheng, Fan Zhang

**Affiliations:** 1 Key Laboratory of Tropical Translational Medicine of Ministry of Education, School of Public Health, Hainan Academy of Medical Sciences, Hainan Medical University, Haikou 571199, China; shilongguo2026@muhn.edu.cn (S.G.); 2404304412@muhn.edu.cn (M.H.);; 2 Haikou Center for Disease Control and Prevention, Haikou 571199, China

**Keywords:** dyslipidemia, middle-aged and older adults, dietary patterns, Southern China

## Abstract

**Objectives:** The objectives of this study were to investigate the association between dietary patterns and dyslipidemia among middle-aged and older adults in Hainan Province and to provide a scientific reference for regional dyslipidemia prevention strategies. **Methods:** A multi-stage cluster random sampling method was used to conduct questionnaire surveys and physical examinations among 4748 middle-aged and older adults from five regions in Hainan Province. Dietary patterns were derived using a food frequency questionnaire and factor analysis. Dietary-pattern scores were categorized into quartiles from lowest (Q1) to highest (Q4) adherence, with Q1 as the reference group. Logistic regression models were used to examine the associations between dietary patterns and four types of dyslipidemia. **Results**: A total of 4748 participants were enrolled, of whom 3321 (69.9%) had dyslipidemia. After adjusting for multiple confounders, compared with Q1, the meat-based dietary pattern at Q4 was positively associated with dyslipidemia (OR = 1.380, *95% CI*: 1.102–1.729). The fruit-and-vegetable pattern was inversely associated with dyslipidemia at Q2 (OR = 0.800, *95% CI*: 0.677–0.945), Q3 (OR = 0.790, *95% CI*: 0.663–0.941), and Q4 (OR = 0.630, *95% CI*: 0.519–0.765). The cooking-oil and wheat pattern was inversely associated with dyslipidemia at Q2 (OR = 0.843, *95% CI*: 0.682–0.980) and Q4 (OR = 0.818, *95% CI*: 0.715–0.993). **Conclusions:** The meat-based dietary pattern was positively associated with dyslipidemia, whereas the fruit-and-vegetable pattern and the cooking-oil and wheat pattern were inversely associated with dyslipidemia. These findings suggest that dietary patterns characterized by higher consumption of fruits, vegetables, and unsaturated fatty acids were inversely associated with dyslipidemia in this population.

## 1. Introduction

China has a vast territory, and dietary patterns vary substantially across regions, shaped by the geographical environment, local food resources, and culinary culture [1]. Unlike the traditional dietary patterns of northern and inland China, which are characterized by higher consumption of wheat-based foods, livestock meat, and high-salt preserved foods, the coastal areas of southern China generally feature rice as a staple food, with abundant consumption of vegetables, fruits, fish and other aquatic products, and poultry. Steaming, poaching, and boiling are commonly preferred cooking methods, resulting in a relatively light and low-salt diet [2]. Located in the tropics, Hainan Province has year-round availability of tropical fruits and vegetables. The dietary habits of its residents therefore exhibit distinct regional characteristics, as confirmed by previous dietary and nutritional surveys [3].

Notably, these dietary characteristics of southern coastal China closely resemble the “Eastern Healthy Dietary Pattern” first proposed in the Dietary Guidelines for Chinese Residents (2022) [4]. This pattern, represented by the traditional diet of southeastern coastal China, is characterized by abundant consumption of vegetables and fruits, frequent consumption of fish, shrimp, and other aquatic products, as well as soy and dairy products, as well as light cooking with limited salt [4]. Residents in these regions have lower rates of obesity and hypertension, lower cardiovascular disease incidence and mortality, and higher life expectancy [4]. Recently, Zhu Shankuan, Yang Yuexin, and colleagues systematically identified and validated an “Eastern Healthy Dietary Pattern” rooted in the eastern coastal region of China using data from the WELL-China cohort. They found that adherence to this pattern was associated with a 22% lower risk of major adverse cardiovascular events and a 17% lower risk of central obesity and explored its potential biological mechanisms through metabolomic and gut microbiome analyses [5].

Dyslipidemia refers to abnormalities in blood lipid metabolism, including elevated levels of total cholesterol (TC), triglycerides (TGs), and low-density lipoprotein cholesterol (LDL-C), or reduced levels of high-density lipoprotein cholesterol (HDL-C). It is a common metabolic disorder [6] and an important risk factor for cardiovascular disease incidence and mortality [7,8,9]. Previous studies have shown that dyslipidemia is the second most prevalent condition among individuals at high risk of cardiovascular disease [10]. Dyslipidemia is influenced not only by biological and genetic factors but is also closely related to dietary factors, and a healthy lifestyle is one of the most effective strategies for managing dyslipidemia [11,12]. However, traditional nutritional epidemiology has primarily focused on the relationships between individual nutrients or selected foods and disease outcomes, making it difficult to capture the overall relationship between dietary intake and health [13]. In contrast, dietary-pattern analysis characterizes the overall combination of foods consumed and therefore provides a more comprehensive representation of actual dietary exposure.

Numerous studies have examined the associations between dietary patterns and dyslipidemia in China and elsewhere [11,12,13], and the Eastern Healthy Dietary Pattern has been identified and associated with cardiometabolic benefits in eastern and coastal China [4,5]. However, it remains unknown whether the locally specific dietary patterns of Hainan, a tropical southern region with distinct food resources, are similarly associated with dyslipidemia. It is also unclear how the a priori Eastern Healthy Dietary Pattern compares with empirically derived Hainan dietary patterns in this population. Furthermore, the tropical dietary characteristics of Hainan residents have not yet been documented using relatively large-scale population-based dietary survey data.

Therefore, based on baseline data from the Hainan Natural Population Chronic Disease Prospective Cohort Study, this study aimed to systematically characterize the major dietary patterns among middle-aged and older adults in Hainan Province and examine their associations with dyslipidemia. The findings are intended to provide epidemiological evidence on the Eastern Healthy Dietary Pattern among middle-aged and older adults in the tropical region of southern China, while also offering locally relevant scientific evidence to inform strategies for the prevention and management of dyslipidemia in this region.

## 2. Materials and Methods

### 2.1. Study Population

Hainan Province has a total population of approximately 10.08 million [14]. The required sample size was estimated based on the prevalence (*P*) of dyslipidemia among adults aged ≥ 35 years in Hainan Province (31.5%) [15]. For this cross-sectional study, the allowable error was set as *d* = 0.1 × *P*, with α = 0.05 and *Z* = 1.96. The sample size was calculated using the formula n = *Z*^2^ × *P*(1 − *P*)/*d*^2^, yielding a minimum sample size of approximately 835 participants. Accounting for an estimated 5% rate of incomplete data, the final sample size was set at 877 participants. This study was conducted from June 2020 to June 2021 using a multi-stage cluster sampling design with random community selection. In the first stage, five survey sites were selected based on the geographic distribution of Hainan Province: Qionghai City (east), Dongfang City (west), Sanya City (south), Haikou City (north), and Qiongzhong County (central). In the second stage, five communities were randomly selected from each of the five regions within the corresponding cities/counties, resulting in a total of 25 communities. After obtaining written informed consent, all eligible residents from the selected communities were enrolled. All participants underwent questionnaire surveys, physical examinations, and biochemical tests. Those with complete data from all three components were included in the analysis.

The inclusion criteria were: (1) residence in Hainan Province for ≥5 years; (2) age 35–74 years; (3) ability to read independently, willingness to participate, and provision of written informed consent; and (4) no prior diagnosis of stroke, coronary heart disease, malignant tumors, chronic obstructive pulmonary disease, dyslipidemia, diabetes, or hypertension before enrollment. Exclusion criteria included a history of psychiatric disorders with long-term use of psychiatric medications, disability, acute or critical illness, or other conditions that might interfere with the study objectives.

Of the 16,559 participants initially enrolled at baseline, only 5433 participants completed all three assessments required for this analysis (questionnaire survey, physical examination, and blood biochemical testing); the remaining participants completed only some of these assessments and thus did not meet the inclusion criteria. After excluding participants with hypertension (n = 167), diabetes mellitus (n = 314), or dyslipidemia (n = 204), a total of 4748 participants were included in the primary analysis (for details, please refer to Figure 1). This study was approved by the Ethics Committee of Hainan Medical College (approval number HYLL-2017-004).

### 2.2. Diagnostic Criteria for Dyslipidemia

Dyslipidemia was defined according to the Chinese Guidelines for Lipid Management (2024) [16]. The diagnostic criteria were as follows: (1) hypercholesterolemia: total cholesterol (TC) ≥ 5.2 mmol/L; (2) hypertriglyceridemia: triglycerides (TG) ≥ 1.7 mmol/L; (3) elevated LDL-C: LDL-C ≥ 3.4 mmol/L; and (4) reduced HDL-C: HDL-C ≤ 1.0 mmol/L. Participants meeting any one of the above criteria were classified as having dyslipidemia [16].

### 2.3. Study Content and Methods

#### 2.3.1. Assessment of General Characteristics

This study was conducted within the framework of the Hainan Natural Population Chronic Disease Prospective Cohort Study (No. 2017YFC0907104) [17,18,19]. A standardized project-specific questionnaire was administered through face-to-face interviews to collect information on participants’ demographic characteristics (age, sex, and residential area), socioeconomic characteristics (occupation and marital status), personal and family medical history, and health-related behaviors, including breakfast consumption, smoking, alcohol consumption, tea consumption, and soup intake. In addition, four lipid profile indicators derived from biochemical examinations were collected to describe the prevalence and distribution of dyslipidemia.

#### 2.3.2. Dietary Assessment

Dietary intake was assessed using a food frequency questionnaire (FFQ) administered face-to-face by trained interviewers, which captured the frequency of consumption of various food groups over the previous year, including wheat-based foods, coarse grains, livestock meat, poultry, aquatic products, and eggs. The dietary-pattern score was developed based on the Eastern Healthy Dietary Pattern (EHDP) proposed in the Dietary Guidelines for Chinese Residents (2022) [4].

The EHDP scoring system comprised 16 components: 10 positive indicators (rice, wheat-based foods, coarse grains, poultry, aquatic products, eggs, fresh vegetables, fresh fruits, nuts, and dairy products) scored from 6 (highest frequency) to 1 (lowest frequency); 5 negative indicators (livestock meat and products, sweets/pastries, pickled vegetables/meat, beverages, and alcohol) scored from 1 (highest frequency) to 6 (lowest frequency); and cooking-oil type, scored as 1 for saturated fatty acids, 3 for mixed, and 6 for unsaturated fatty acids. Each of the 16 components was scored from 1 to 6, yielding a total EHDP score ranging from 16 to 96. A higher total score indicated greater adherence to a healthier dietary pattern. This a priori score was analyzed separately from the four factor-analysis-derived dietary patterns [20]. The dietary patterns subsequently derived by factor analysis were based on the above scoring system.

#### 2.3.3. Physical Examination

Physical examinations included measurements of height, body weight and blood pressure. Before data collection, all investigators received standardized training from the research team and used calibrated instruments that met international measurement standards. Each participant was measured twice using standardized procedures, and the mean of the two measurements was used for analysis. All measurements were recorded to one decimal place.

#### 2.3.4. Blood Lipid Measurements

Fasting venous blood samples were collected from participants. Sample collection, processing, storage, and biochemical analyses were performed in accordance with standardized laboratory protocols. Four lipid parameters, namely total cholesterol (TC), triglycerides (TGs), low-density lipoprotein cholesterol (LDL-C), and high-density lipoprotein cholesterol (HDL-C), were measured using an automated biochemical analyzer 7600-110 (Hitachi, Ibaraki, Japan).

### 2.4. Statistical Analysis

A database was established using Microsoft Excel 2016, and data entry, verification, and cleaning were performed using a double-entry procedure. All statistical analyses were conducted using SPSS version 26.0. Dietary patterns were derived using factor analysis with principal component extraction. Factor scores were calculated, and dietary patterns were identified based on factor loadings of food groups. Dietary-pattern scores were divided into quartiles (Q1–Q4), with the lowest quartile (Q1) used as the reference group. The EHDP score, as a predefined score rather than a factor-analysis-derived pattern, was similarly divided into quartiles for logistic regression analysis. The chi-square test was used for univariate analyses. Covariates were selected based on a directed acyclic graph informed by the previous literature [15,17,18,19] and biological plausibility. To account for potential within-community correlation, logistic regression models were fitted using generalized estimating equations with a binomial distribution and logit link, specifying community as the clustering unit. An independent working correlation structure and a robust sandwich covariance estimator were used to obtain community-clustered robust standard errors for all regression models. We used a staged adjustment strategy: Model 1 adjusted for demographic factors (age, sex, marital status, occupation); Model 2 additionally adjusted for lifestyle factors (smoking, alcohol consumption, tea consumption, breakfast consumption); and Model 3 further adjusted for BMI, hypertension, and diabetes. The fully adjusted model (Model 3) is presented in Figure 2. To assess linear trends across quartiles, the median dietary-pattern score within each quartile was assigned to all participants in that quartile. The resulting variable was entered as a continuous term in the multivariable logistic regression model, with adjustment for the same covariates as in the quartile-based analyses. The *p* value for the coefficient of this variable was reported as the *P* for trend. All statistical tests were two-sided, and the significance level was set at α = 0.05. Because multiple dietary-pattern quartiles and lipid outcomes were examined, no formal multiplicity adjustment was applied, consistent with the exploratory nature of this cross-sectional study; isolated associations, particularly those with *p* values near 0.05, should therefore be interpreted cautiously.

## 3. Results

### 3.1. Basic Characteristics of the Study Population

A total of 4748 participants were included in this study, comprising 2327 males (49.0%) and 2421 females (51.0%). The median age was 47 years (interquartile range: 41–55 years). Among the participants, 2657 (58.7%) had hypercholesterolemia, 1190 (25.1%) had hypertriglyceridemia, 1526 (32.1%) had elevated LDL-C, and 176 (3.7%) had reduced HDL-C. Overall, 3321 participants (69.9%) were identified as having dyslipidemia.

### 3.2. Classification of Dietary Patterns

Dietary patterns were derived using exploratory factor analysis (EFA) based on the 14 food groups assessed by the FFQ. Prior to factor analysis, all frequency scores were standardized to ensure comparability across food groups. Principal component analysis (PCA) was employed as the initial extraction method, followed by orthogonal varimax rotation to maximize the interpretability of the derived factors while maintaining their mutual independence, which is desirable when the factors are subsequently used as separate exposures in regression models. Principal component analysis was used for factor extraction. The Kaiser–Meyer–Olkin (KMO) value was 0.829, and Bartlett’s test of sphericity was statistically significant (*p* < 0.01), indicating that the data were suitable for factor analysis. Four factors with eigenvalues > 1.0 were extracted, accounting for 50.01% of the total variance. The four major dietary patterns and their factor loadings are presented in Table 1. Food groups with absolute factor loadings > 0.30 were retained for each pattern, and the patterns were named according to the food groups that contributed most strongly to each factor.

Based on factor scores, the four identified patterns were named the modern fast-food pattern, meat-based pattern, fruit-and-vegetable pattern, and cooking-oil and wheat pattern. Factor scores were divided into quartiles (Q1–Q4) from lowest to highest, with Q1 used as the reference group. Higher factor scores indicated greater adherence to the corresponding dietary pattern. The EHDP was treated as a separate exposure variable based on its predefined scoring criteria, distinct from the four patterns derived through factor analysis.

### 3.3. Associations Between Dietary Patterns and Dyslipidemia

As shown in Table 2, the modern fast-food pattern was significantly associated with reduced HDL-C, elevated LDL-C, and dyslipidemia. The meat-based pattern showed a significant association with elevated LDL-C. The fruit-and-vegetable pattern was significantly associated with hypertriglyceridemia. The cooking-oil and wheat pattern was significantly associated with dyslipidemia. The Eastern Healthy Dietary Pattern was significantly associated with reduced HDL-C and elevated LDL-C (all *p* < 0.05).

### 3.4. Multivariable Logistic Regression Analysis of Dietary Patterns and Dyslipidemia

Dietary-pattern scores of middle-aged and older adults in Hainan Province were divided into quartiles. The models were adjusted for potential confounders, including breakfast consumption, marital status, occupation, tea consumption, age, years of employment, diabetes, hypertension, obesity (defined by BMI), sex, alcohol consumption, and smoking status. Using Q1 as the reference, the highest quartile (Q4) of the meat-based pattern was associated with higher odds of dyslipidemia (OR = 1.380, *95% CI*: 1.102–1.729). In contrast, the fruit-and-vegetable pattern at Q2 (OR = 0.800, *95% CI*: 0.677–0.945), Q3 (OR = 0.790, *95% CI*: 0.663–0.941), and Q4 (OR = 0.630, *95% CI*: 0.519–0.765) was associated with lower odds of dyslipidemia. Similarly, the cooking-oil and wheat pattern at Q2 (OR = 0.843, *95% CI*: 0.682–0.980) and Q4 (OR = 0.818, *95% CI*: 0.715–0.993) showed significant inverse associations with dyslipidemia. For hypertriglyceridemia, the meat-based pattern at Q2 (OR = 1.338, *95% CI*: 1.097–1.632), Q3 (OR = 1.403, *95% CI*: 1.126–1.749), and Q4 (OR = 1.578, *95% CI*: 1.212–2.053) was associated with higher odds. Conversely, the fruit-and-vegetable pattern at Q2 (OR = 0.787, *95% CI*: 0.651–0.951), Q3 (OR = 0.710, *95% CI*: 0.581–0.866), and Q4 (OR = 0.493, *95% CI*: 0.392–0.620), as well as the cooking-oil and wheat pattern at Q4 (OR = 0.733, *95% CI*: 0.593–0.905), were associated with lower odds. For reduced HDL-C, the modern fast-food pattern at Q4 (OR = 3.799, *95% CI*: 1.731–8.334) and the meat-based pattern at Q4 (OR = 2.934, *95% CI*: 1.701–5.064) were associated with higher odds. The Eastern Healthy Dietary Pattern at Q2 (OR = 0.385, *95% CI*: 0.228–0.651), Q3 (OR = 0.206, *95% CI*: 0.103–0.413), and Q4 (OR = 0.066, *95% CI*: 0.027–0.163) was associated with lower odds. For elevated LDL-C, the modern fast-food pattern at Q2 (OR = 1.322, *95% CI*: 1.045–1.672) and the meat-based pattern at Q4 (OR = 1.286, *95% CI*: 1.011–1.637) were associated with higher odds. All the above associations were statistically significant (*p* < 0.05). No significant associations were observed for the remaining dietary patterns and outcomes. Throughout this manuscript, effect sizes and 95% confidence intervals are emphasized over *p* values alone.

Trend analyses showed that higher adherence to the fruit-and-vegetable pattern and the cooking-oil and wheat pattern were inversely associated with overall dyslipidemia (*P* for trend < 0.001 and *P* for trend = 0.003, respectively). An inverse trend was also observed for the EHDP score (*P* for trend = 0.021), although none of the individual EHDP quartiles differed significantly from Q1. For individual lipid abnormalities, significant trends were observed for the modern fast-food pattern with reduced HDL-C and elevated LDL-C; the meat-based pattern with hypertriglyceridemia and elevated LDL-C; the fruit-and-vegetable pattern with hypertriglyceridemia; the cooking-oil and wheat pattern with hypertriglyceridemia; and the EHDP score with reduced HDL-C (all *P* for trend < 0.05; Figure 2).

## 4. Discussion

### 4.1. Main Findings and Comparison with Previous Studies

In this study, four dietary patterns were identified among middle-aged and older adults in Hainan Province: the modern fast-food pattern, meat-based pattern, fruit-and-vegetable pattern, and cooking-oil and wheat pattern. The dietary patterns observed in Hainan Province differed considerably from those in most other regions of China, but were broadly similar to those reported in the southeastern coastal regions, where the Eastern Healthy Dietary Pattern has been described [2].

Trend analyses supported inverse associations of the fruit-and-vegetable and cooking-oil and wheat patterns with overall dyslipidemia, with the most consistent gradient observed for the fruit-and-vegetable pattern. Greater adherence to the meat-based pattern showed significant positive trends with hypertriglyceridemia and elevated LDL-C; however, despite higher odds of overall dyslipidemia in Q4, its linear trend was not significant. The EHDP score showed a strong inverse trend with reduced HDL-C, whereas its inverse trend with overall dyslipidemia should be interpreted cautiously because no individual quartile comparison was significant. These statistical trends should not be considered evidence of causal dose–response relationships given the cross-sectional design.

The meat-based pattern was associated with 38% higher odds of dyslipidemia (OR = 1.380, *95% CI*: 1.102–1.729), corresponding to a moderate effect size that may have clinical relevance given the high prevalence of dyslipidemia in this population (69.9%). This pattern was characterized by high intakes of poultry meat and cholesterol, both of which have been linked to adverse lipid profiles in previous experimental and clinical studies [21,22]. For instance, saturated fatty acids have been shown to activate the sterol regulatory element-binding protein 1c (SREBP-1c) pathway in the liver, thereby promoting triglyceride synthesis and inhibiting LDL receptor activity, which may lead to LDL-C accumulation. Likewise, dietary cholesterol has been reported to upregulate acetyl-CoA acetyltransferase 2 (ACAT2), facilitating the incorporation of cholesterol esters into very-low-density lipoprotein (VLDL) and thereby increasing circulating lipid levels [21,22].

In contrast, the fruit-and-vegetable pattern was associated with a 37% reduction in dyslipidemia odds at Q4 (OR = 0.630, *95% CI*: 0.519–0.765), representing a relatively strong inverse association. The fruit-and-vegetable pattern was characterized by higher intakes of fresh fruits and vegetables, which are rich sources of dietary fiber, vitamins, minerals, and antioxidants. The components may contribute to lipid regulation, as suggested by previous studies [23]. Zhang et al. [24] reported that higher consumption of high-quality fruits and vegetables was associated with improved biomarkers and a lower prevalence of multiple chronic conditions, including dyslipidemia. Similarly, the cooking-oil and wheat pattern, characterized primarily by cooking oils (loading 0.753) and wheat products (loading 0.545), was inversely associated with dyslipidemia. Because fatty-acid composition was not quantitatively measured, any mechanism involving unsaturated fatty acids is discussed only as a possible explanation, not as a directly assessed exposure. Previous evidence has suggested that unsaturated fatty acids may favorably modulate lipid metabolism by reducing TG and LDL-C and increasing HDL-C, which may in turn lower the risk of cardiovascular disease [25].

### 4.2. Implications for the Eastern Healthy Dietary Pattern

The present findings are directionally consistent with the core principles of the Eastern Healthy Dietary Pattern proposed in the Dietary Guidelines for Chinese Residents (2022) [4], as well as with the results reported by Yang Yuexin, Zhu Shankuan, and colleagues, who identified the Eastern Healthy Dietary Pattern in eastern Chinese populations and observed its potential benefits for cardiometabolic health [5]. The current study extends this line of evidence by applying the framework to a population in the tropical region of southern China, an area not previously covered in this context.

In this cross-sectional analysis, dietary patterns rich in vegetables, fruits, and oils rich in unsaturated fatty acids were associated with lower odds of dyslipidemia; whether promoting such dietary changes would improve blood lipid profiles should be tested in future intervention studies. A “tropical healthy dietary pattern” may therefore merit evaluation in future dietary interventions.

### 4.3. Limitations

Although this study provides population-based evidence on dietary patterns and dyslipidemia in Hainan, several limitations should be considered when interpreting the findings. First, dietary intake was assessed using an FFQ that recorded consumption frequency rather than quantitative intake. As a result, energy and nutrient intakes could not be estimated, and the FFQ has not been formally validated or tested for reproducibility in this population. These limitations may have led to dietary-pattern misclassification and residual confounding. Second, the cross-sectional design precludes causal inference. Recall bias is possible, and reverse causation cannot be ruled out, as participants may have modified their diets after learning of elevated lipid levels. Third, multiple comparisons were made across five dietary exposures and five lipid outcomes, and no formal adjustment for multiplicity was applied; findings with *p* values near 0.05 may therefore be due to chance. The derivation of dietary patterns also involves subjective decisions inherent in factor analysis. Fourth, selection bias may have arisen from the reduction in the number of participants from 16,559 to 4748, largely because many participants lacked complete questionnaires, a physical examination, or biochemical data. Finally, the findings may not be generalizable beyond middle-aged and older adults in Hainan. Future longitudinal studies in diverse populations are warranted.

## 5. Conclusions

In this population-based cross-sectional study, four distinct dietary patterns were identified among middle-aged and older adults in Hainan Province. The meat-based dietary pattern was positively associated with dyslipidemia, whereas the fruit-and-vegetable pattern and the cooking-oil and wheat pattern were inversely associated with dyslipidemia. These findings suggest that dietary patterns characterized by higher consumption of fruits, vegetables, and unsaturated fatty acids may be associated with lower odds of dyslipidemia in this population. However, owing to the cross-sectional design, causal relationships cannot be established. Future longitudinal studies are warranted to confirm these associations and to clarify the temporal relationships between dietary patterns and dyslipidemia.

## Figures and Tables

**Figure 1:**
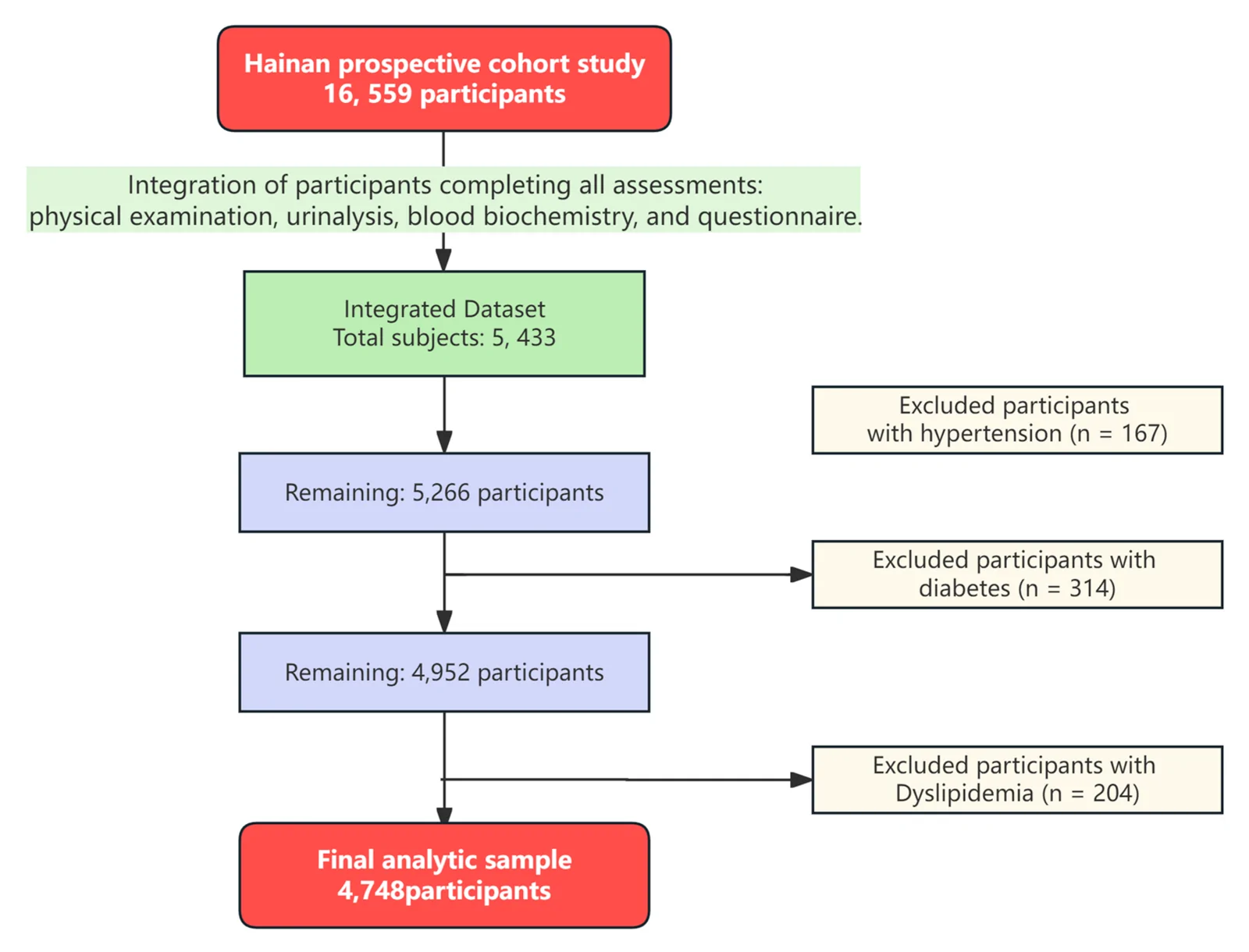
Analysis flowchart.

**Figure 2:**
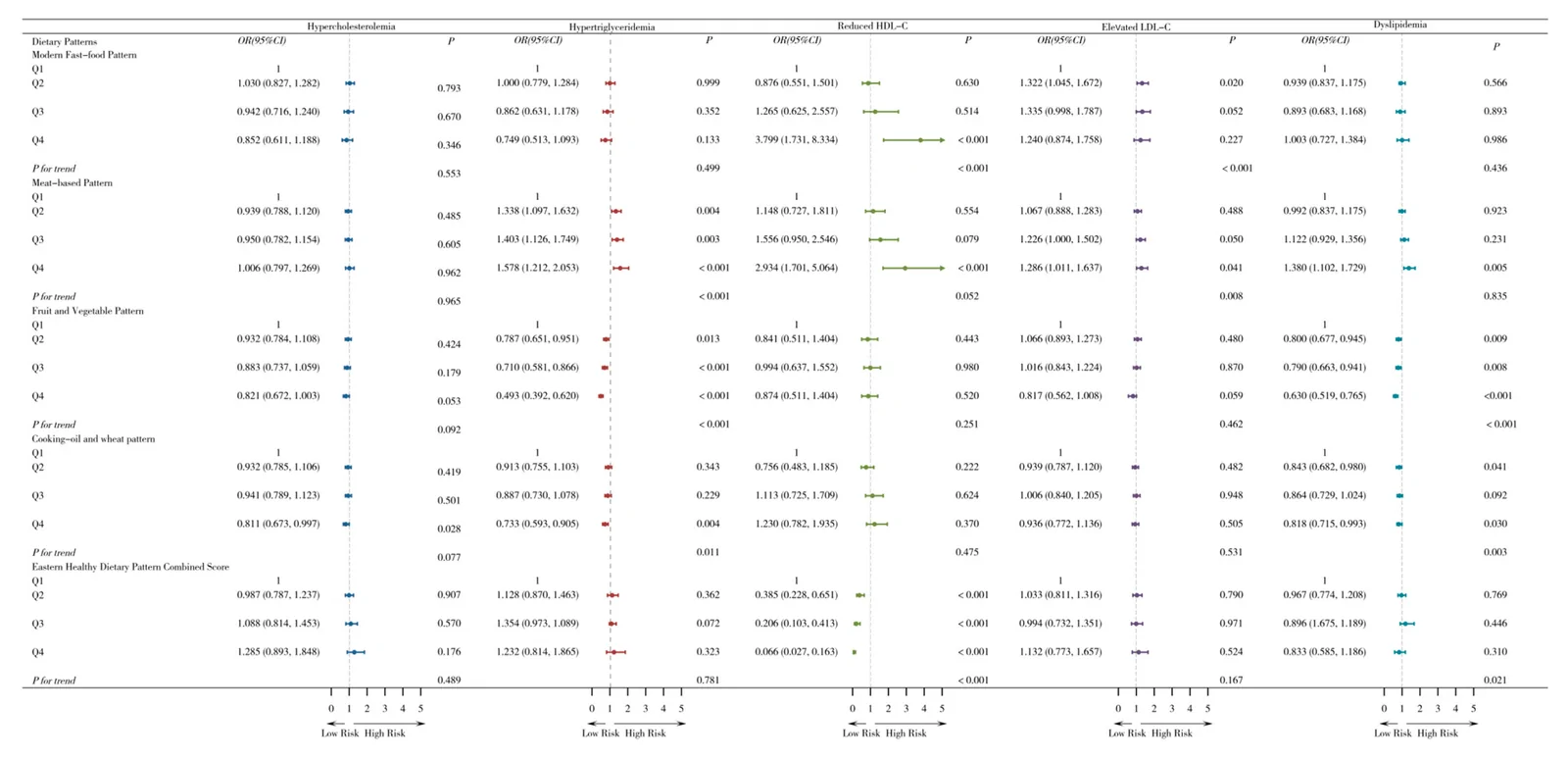
Multivariable logistic regression analysis of dietary patterns and dyslipidemia, including tests for linear trend: adjusted for age, sex, marital status, occupation, smoking, alcohol consumption, tea consumption, breakfast consumption, BMI, hypertension, and diabetes (Model 3).

**Table 1:** Factor loadings of dietary patterns among middle-aged and older adults in Hainan Province.

Food Categories	Factor Loading			
	Modern Fast-Food Pattern	Meat- Based Pattern	Fruit-and-Vegetable Pattern	Cooking-Oil and Wheat Pattern
Rice	0.595	—	0.306	—
Wheat Products	−0.323	—	—	0.545
Coarse Grains	−0.612	—	—	—
Sweets	0.636	—	—	—
Red Meat	—	−0.726	—	—
Poultry Meat	—	0.797	—	—
Fish and Shellfish	—	0.623	—	—
Eggs	—	0.389	0.355	—
Vegetables	0.455	—	0.429	—
Fruits	—	—	0.754	—
Nuts and Seeds	−0.741	—	—	—
Pickled Vegetables	0.734	—	—	—
Milk and Dairy	−0.648	—	—	—
Beverages	0.691	—	—	—
Alcohol	—	—	0.400	—
Cooking Oils	—	—	—	0.753

Notes: Only food items with absolute factor loadings > 0.30 are presented. The Eastern Healthy Dietary Pattern (EHDP) is a predefined score and is presented separately from the four factor-analysis-derived patterns.

**Table 2:** Univariate analysis of dyslipidemia among middle-aged and older adults in Hainan Province [n (%)].

Characteristic	Hypercholesterolemia		Hypertriglyceridemia		Reduced HDL-C		Elevated LDL-C		Dyslipidemia	
	N (%)	χ^2^	N (%)	χ^2^	N (%)	χ^2^	N (%)	χ^2^	N (%)	χ^2^
Modern Fast-food Pattern										
Q1	685 (25.8)	0.446	300 (25.2)	0.681	81 (46.0)	42.573 ^b^	320 (21.0)	19.855 ^b^	809 (24.4)	11.921 ^a^
Q2	680 (25.6)		307 (25.8)		30 (17.0)		393 (25.8)		820 (24.7)	
Q3	667 (25.1)		302 (25.4)		24 (13.6)		409 (26.8)		815 (24.5)	
Q4	625 (23.5)		281 (23.6)		41 (23.4)		404 (26.4)		877 (26.4)	
Meat-based Pattern										
Q1	673 (25.3)	0.985	265 (22.3)	7.393	47 (26.7)	3.599	350 (23.0)	7.839 ^a^	824 (24.8)	3.329
Q2	657 (24.7)		320 (26.9)		38 (21.6)		370 (24.2)		823 (24.8)	
Q3	665 (25.1)		312 (26.2)		39 (22.2)		403 (26.4)		819 (24.7)	
Q4	662 (24.9)		293 (24.6)		52 (29.5)		403 (26.4)		855 (25.7)	
Fruit-and-Vegetable Pattern										
Q1	691 (26.0)	2.917	349 (29.3)	25.642 ^b^	55 (31.3)	4.293	369 (24.2)	6.987	860 (25.9)	8.202 ^b^
Q2	662 (24.9)		301 (25.3)		36 (20.4)		404 (26.5)		837 (25.2)	
Q3	656 (24.7)		298 (25.0)		44 (25.0)		400 (26.2)		827 (24.9)	
Q4	648 (24.4)		242 (20.4)		41 (23.3)		353 (23.1)		797 (24.0)	
Cooking-oil and Wheat pattern										
Q1	678 (25.4)	3.675	317 (26.6)	7.209	54 (30.7)	5.088	378 (24.8)	1.293	857 (25.8)	5.365
Q2	655 (24.7)		299 (25.1)		33 (18.8)		369 (24.2)		833 (25.1)	
Q3	671 (25.3)		302 (25.4)		44 (25.0)		394 (25.8)		825 (24.8)	
Q4	653 (24.6)		272 (22.9)		45 (25.5)		385 (25.2)		806 (24.3)	
Eastern Healthy Dietary Pattern (EHDP) Score										
Q1	695 (26.2)	2.461	301 (25.3)	3.344	83 (47.2)	45.206 ^b^	330 (21.6)	19.235 ^b^	813 (24.5)	3.201
Q2	654 (24.6)		296 (24.9)		38 (21.6)		382 (25.0)		825 (24.8)	
Q3	653 (24.5)		319 (26.8)		33 (18.7)		384 (25.2)		831 (25.0)	
Q4	655 (24.7)		274 (23.0)		22 (12.5)		430 (28.2)		852 (25.7)	
Occupation										
Not Currently Employed	303 (11.40)	6.471	125 (10.5)	19.014 ^b^	65 (36.9)	139.655 ^b^	110 (7.2)	32.000 ^b^	377 (11.4)	15.798 ^a^
Freelancer	53 (1.99)		22 (1.9)		5 (2.8)		20 (1.3)		63 (1.9)	
Blue-collar Worker	1379 (51.90)		565 (47.5)		66 (37.5)		831 (54.5)		1755 (52.8)	
White-collar Worker	790 (29.73)		406 (34.1)		35 (19.9)		472 (30.9)		953 (28.7)	
Others	132 (4.97)		72 (6.0)		5 (2.9)		93 (6.1)		173 (5.2)	
Characteristic	Hypercholesterolemia		Hypertriglyceridemia		Reduced HDL-C		Elevated LDL-C		Dyslipidemia	
	n (%)	*χ* ^2^	n (%)	*χ* ^2^	n (%)	*χ* ^2^	n (%)	*χ* ^2^	n (%)	*χ* ^2^
BMI										
Underweight	100 (3.8)	31.236 ^b^	25 (2.1)	181.981 ^b^	14 (8.0)	6.909	27 (1.8)	121.914 ^b^	122 (3.7)	75.035 ^b^
Normal Weight	1279 (48.1)		435 (36.6)		77 (43.8)		651 (42.7)		1577 (47.5)	
Overweight and Obese	1278 (48.1)		730 (61.3)		85 (48.2)		848 (55.6)		1622 (48.8)	
Hypertension Status										
Hypertension	1060 (39.9)	49.745 ^b^	584 (49.1)	126.025 ^b^	85 (48.3)	12.704 ^b^	620 (40.6)	26.320 ^b^	1326 (39.9)	96.039 ^b^
Normotension	1597 (60.1)		606 (50.9)		91 (51.7)		906 (59.4)		1995 (60.1)	
Diabetes										
Yes	238 (8.96)	16.164 ^b^	159 (13.4)	75.755 ^b^	43 (24.4)	73.723 ^b^	117 (7.7)	0.035	303 (9.1)	38.606 ^b^
No	2419 (91.04)		1031 (86.6)		133 (75.6)		1409 (92.3)		3018 (90.9)	
Gender										
Male	1363 (51.30)	15.032 ^b^	817 (68.7)	252.547 ^b^	100 (56.8)	4.461	880 (57.7)	67.283 ^b^	1761 (53.0)	71.317 ^b^
Female	1294 (48.70)		373 (31.4)		76 (43.2)		646 (42.3)		1560 (47.0)	
Age Group										
35–45	841 (42.2)	70.783 ^b^	440 (37.0)	15.716 ^b^	27 (15.4)	333.795 ^b^	582 (38.2)	158.125 ^b^	1246 (37.5)	91.250 ^b^
46–55	916 (54.9)		455 (38.2)		30 (17.0)		668 (43.8)		1242 (37.4)	
56–65	342 (61.7)		152 (12.8)		24 (13.6)		210 (13.7)		421 (12.7)	
66–74	395 (74.2)		143 (12.0)		95 (54.0)		66 (4.3)		412 (12.4)	
Breakfast Consumption										
Yes	2154 (81.2)	2.534	940 (79.3)	1.506	100 (56.8)	65.013 ^b^	1282 (84.3)	18.849 ^a^	2676 (80.7)	0.003
No	498 (18.8)		246 (20.7)		76 (43.2)		238 (15.7)		638 (19.3)	
Marital Status										
Married	2329 (87.7)	2.626	1034 (86.9)	3.144	133 (75.6)	28.875 ^b^	1384 (90.7)	11.038 ^b^	2923 (88.0)	1.921
Others	328 (12.3)		156 (13.1)		43 (24.4)		142 (9.3)		398 (12.0)	
Smoking Status										
Yes	835 (31.4)	3.206	546 (45.9)	183.568 ^b^	83 (47.2)	24.412 ^b^	526 (34.5)	17.997 ^b^	1098 (33.1)	38.467 ^b^
No	1822 (68.6)		644 (54.1)		93 (52.8)		1000 (65.5)		2223 (66.9)	
Tea Consumption										
Yes	1816 (68.4)	2.005	902 (75.8)	50.795 ^b^	137 (77.8)	8.880 ^a^	1085 (71.1)	12.729 ^b^	2286 (68.8)	7.860 ^a^
No	841 (31.6)		288 (24.2)		39 (22.2)		441 (28.9)		1035 (31.2)	
Alcohol Consumption										
Yes	1605 (60.4)	2.784	831 (69.8)	73.359 ^b^	123 (69.9)	8.400 ^a^	937 (61.4)	3.795	2040 (61.4)	18.952 ^b^
No	1052 (39.6)		359 (30.2)		53 (30.1)		589 (38.6)		1281 (38.6)	

Notes: ^a^ *p* < 0.05; ^b^ *p* < 0.001.

## Data Availability

The data presented in this study are available upon reasonable request from the corresponding author. Due to privacy and ethical restrictions, the data are not publicly available.

## Abbreviations

The following abbreviations are used in this manuscript:

Abbreviation	Full Term
TC	Total Cholesterol
TGs	Triglycerides
LDL-C	Low-Density Lipoprotein Cholesterol
HDL-C	High-Density Lipoprotein Cholesterol
OR	Odds Ratio
CI	Confidence Interval
FFQ	Food Frequency Questionnaire
EHDP	Eastern Healthy Dietary Pattern
KMO	Kaiser–Meyer–Olkin
BMI	Body Mass Index
SPSS	Statistical Package for the Social Sciences
